# Muscle Tone Regulation and Bruxism in Chronic Stress: Pathophysiological Links to Tooth Fractures and Dental Hard Tissue Pathology

**DOI:** 10.3390/medsci14020320

**Published:** 2026-06-15

**Authors:** Valekh Ashyrov, Maria Blagodatskikh, Olga Panferova, Irina Vineyard, Lucas Alves Sarmento Pires, Tatiana Zharikova, André Pontes-Silva, Yury Zharikov

**Affiliations:** 1 Department of Human Anatomy and Histology, Sechenov First Moscow State Medical University (Sechenov University), 125009 Moscow, Russia; ashyrov04@mail.ru (V.A.); maria.blagodatskikh@mail.ru (M.B.); oi.panferova@mail.ru (O.P.);; 2 Morphology Department, Fluminense Federal University—UFF, Niterói 24210-201, Brazil; 3 Postgraduate Program in Physical Education, NERD Research Group, Federal University of Maranhão, São Luís 65080-805, Brazil

**Keywords:** muscle tone, bruxism, masticatory muscles, autonomic nervous system, tooth wear, tooth fractures

## Abstract

Anxiety disorders and chronic stress are the most common types of mental disorder. According to the WHO, more than 359 million people worldwide suffered from these conditions in 2021. The function of mastication and the masticatory muscles undergo significant changes under the influence of a disturbed psychoemotional state. This manifests as their parafunctional activity, accompanied by increased tone and damage to elements of the dentofacial system, including increased tooth wear, chipping, cracks, and fractures. Attention to this problem is growing annually among researchers in both dental and neurological fields. This is evidenced by a wide range of therapeutic and preventive interventions aimed at correcting chronic stress, muscle hypertonia, and pathology of the dentofacial system. Despite the aforementioned measures, it is often only possible to slow down the pathological process rather than completely resolve it. This is because knowledge regarding the biology and pathophysiology of how chronic stress affects muscle activity remains limited. Understanding such mechanisms and establishing precise interrelationships could help identify targets for effective therapeutic interventions and eliminate the problem. This review of the literature systematizes information on how chronic stress and various autonomic stimuli affect changes in the functional activity of the masticatory muscles and the pathology of hard dental tissues.

## 1. Introduction

Today, mental disorders are among the most common nosologies in the population, with the number of people affected approaching 1 billion worldwide. Anxiety disorders and chronic stress are the most common types of mental disorders. The WHO reports that over 359 million people worldwide have been diagnosed with these conditions [1]. It is known that disorders of the psychoemotional state can have pleiotropic effects on the human body and affect a number of physiological processes and normal organ functions [2,3], such as the function of the masticatory muscles, disorders of which have become a serious problem for the dental community because they alter the dental hard tissues and elements of the temporomandibular joint. A number of relationships between anxiety disorders, chronic stress, and abnormalities in the tone of the masseter muscles have already been identified [4]. At the same time, studies to date have more often examined individual components of this condition without addressing the entirety of the pathological processes involved. This leads to a limited understanding of the interactions among all links in the chain, from central nervous system disorders to pathology of the hard dental tissues. Therefore, the characteristics of masticatory muscle tone under chronic stress are important for interdisciplinary research and for understanding the above-mentioned mechanisms [5]. This work aims to provide a comprehensive review of the various mechanisms that maintain the tone of the masseter muscles in normal and pathological conditions, the impact of tone abnormalities on the dental hard tissues, and the measures that can be taken to prevent these abnormalities.

## 2. Physiological and Anatomical Basis for Maintaining Normal Muscle Tone in the Masseter Muscles

Muscle tone is the background level of muscle tension at rest, manifested as resistance to stretching. It includes a passive viscoelastic component, determined by the structural properties of tissues, and an active component associated with low-level, sustained activation of motor units [6,7]. The intrinsic tone of the masticatory muscles is a key mechanism for maintaining the position of the mandible at rest and is ensured by a number of reflex actions associated with the structural and innervation features of this muscle group. Moreover, the activity of the masticatory muscles changes depending on the functional load: V.F. Ferrario noted that, at rest, the potentials of the temporalis and masseter muscles averaged 1.9 μV and 1.4 μV, whereas during voluntary contraction (teeth clenching), the average maximum potential values were 181.9 μV (temporalis muscle) and 216.2 μV (masseter muscle) in men, and 161.7 μV (temporalis muscle) and 156.8 μV (masseter muscle) in women [8].

One of the main components of muscle tone is the myotatic reflex, associated with the functioning of intrafusal muscle fibers that make up muscle spindles. This reflex provides the necessary tension of the masticatory muscles to maintain an optimal position of the mandible in space. Possessing an abundance of branching nerve endings and attachment to connective tissue layers between extrafusal fibers, intrafusal fibers represent an important link in signaling the degree of muscle contraction [9,10]. An important feature of the masticatory muscles is the presence of many intrafusal fibers in a single spindle—up to 36 units, according to an immunohistochemical study by Eriksson et al. which is several times higher than that found in other muscles and indicates their significant role in proprioception in this region [11].

Their mechanism of action is realized when the masticatory muscles are stretched under the force of gravity and there is a decrease in their tone, which is accompanied by an increase in the length of the extrafusal muscle fibers and irritation of the nerve endings on the intrafusal fibers. Afferentation from these receptors in the central nervous system (CNS) is carried along the fibers of the trigeminal nerve to its sensory mesencephalic nucleus (MesV) [12]. This is where neurons are located that activate cells of the motor nuclei and influence the contraction of the masticatory muscles. At the same time, there is a group of cells that inhibit neurons of the motor nuclei of the trigeminal nerve, which helps control excessive jaw closure [13].

An important feature of MesV is its localization within the central nervous system, rather than in the ganglion, which allows it to synaptically regulate the activity of neurons in this formation through the influence of other central nervous system structures [14,15]. Additionally, it is worth noting that MesV axons have connections with the reticular formation, vestibular nuclei of the pons, and the thalamus, from whose neurons impulses propagate to the primary somatosensory and insular cortex [16,17,18]. Thus, the presence of a pathological process in these components of the central nervous system can lead to impaired muscle tone and masticatory function. There is also evidence of a connection between muscle spindles and the supratrigeminal nucleus of the trigeminal nerve, which sends signals to the ventral posteromedial nucleus of the thalamus via the dorsal trigeminothalamic tract [19]. This structure regulates the tone of the masticatory muscles and bilateral jaw closure, and also has a pool of connections with various areas of the brain that regulate autonomic and behavioral functions [20,21]. When discussing the cortical representation of this pathway, it is worth highlighting the primary and secondary somatosensory cortex and the granular cortex of the insula [22]. Some researchers note connections with the cerebellar cortex, the significance of which remains poorly understood [23]. These areas also have connections with elements of the limbic system, which links various emotional and behavioral responses to changes in muscle tone in the maxillofacial region [24]. Thus, the resting muscle tone of the masticatory muscles is ensured by the functioning of a multicomponent reflex arc and can be regulated by influences from various parts of the central nervous system, synaptically connected to the links of this chain.

Special attention should also be given to the nerve endings of the periodontal ligament, which play an important role in regulating the tone of the masticatory muscles [25]. As is known, the periodonto-muscular reflex arc, which controls the force of chewing pressure and limits excessive muscle tension due to the inhibitory effect on the motor neurons of the motor nuclei of the trigeminal nerve, begins with the mechanoreceptors of the periodontium [26,27]. This function makes it possible to limit excessive masticatory pressure and reduce the wear of hard dental tissues. On the other hand, this mechanism allows for a rapid release of masticatory pressure when a hard food fragment is encountered among soft food during chewing. Such a mechanism helps reduce the occurrence of dental injuries and chipping. These data are confirmed by the study of B. Bonte et al., which demonstrates a reflex decrease in muscle pressure upon the activation of receptors of the preserved periodontal ligament in comparison with titanium implants, which are devoid of periodontal nerve endings [28]. Based on this, it is worth noting that a violation of the control of muscle contractions by the periodontium can lead to significant dysregulation of chewing pressure. This is confirmed by various studies showing changes in periodontal nerve endings in patients with bruxism [29,30].

## 3. The Effect of the Autonomic Nervous System on Muscle Tone and the Development of Its Pathology

Over the past several decades, a significant amount of data has accumulated linking the development of masticatory muscle disorders with dysfunction in various parts of the nervous system [31,32,33]. Some of the most interesting structures to consider, in our opinion, are the components of the autonomic nervous system, which exert pleiotropic effects on various processes both in the brain and throughout the body as a whole. Although autonomic nerve trunks cannot directly innervate striated masticatory muscle fibers, they can modulate their state by altering vascular tone and regulating neuronal activity in various parts of the myotatic reflex arc [34].

The hypothalamus, as the highest autonomic center, is directly involved in regulating the functioning of the trigeminal nuclei. McGregor R. et al. report in their study the presence of a direct projection between the hypothalamic nuclei and the motor nuclei of the trigeminal nerve, revealed by retrograde staining [35]. Yasui Y., studying the architecture of this projection, supplements it with data on the connections of the dorsomedial and lateral hypothalamus with the motor nuclei and MesV of the trigeminal nerve [36]. By enhancing glutamatergic transmission, the activity of MesV neurons increases, which may heighten sensitivity to changes in muscle tone. This allows for the indirect modulation of the regulation of masticatory muscle tone. Thus, the hypothalamus, through its own structures, can directly modulate the activity of the trigeminal nucleus system and influence the tone of the masticatory muscles and masticatory function in general.

There are also pathways through which the hypothalamus performs its functions through other structures. Thus, Kagan R. et al. in their work experimentally demonstrate the presence of projections between the hypothalamus and the ventral posteromedial nuclei of the thalamus, which receive nociceptive and proprioceptive signals from the fibers of the trigeminal nerve. The positive immunostaining of axonal arborizations found there against vesicular glutamate transporters (VGLUT2) indicates an activating influence of the hypothalamus on neurons of the ventral posteromedial nucleus of the thalamus. This creates conditions for the regulation of proprioception and pain sensitivity in the maxillofacial region by the autonomic centers of perception and processing [37]. Studying other related structures, Mavanji V. et al. revealed increased activity of the primary somatosensory cortex and hyperactivity of the masticatory muscles after intracerebral injection of Orexin-A, a hypothalamic neuropeptide that regulates various brain functions (including the regulation of muscle tone) [38]. Orexin-A mediates its effects through G-protein-coupled receptors and can induce sustained excitation of motor neurons. The study demonstrates the role of the formation of projections from orexin neurons of the hypothalamus to areas of the brain involved in the regulation of muscle tone. Literature data show that the hypothalamus is an important center supporting the function of the masticatory muscles by influencing various CNS structures that ensure their functioning [39,40].

## 4. The Influence of the Sympathetic Division

Continuing to examine the relationship between the effects of the autonomic nervous system and the activity of the masticatory muscles, it is worth separately examining the functions of each component of this division. The sympathetic division is involved in maintaining general homeostasis and prepares the body for a “fight-or-flight” response caused by appropriate exogenous factors [41]. Despite the fact that most of the major sympathetic centers are concentrated outside the brain, sympathetic innervation has a pool of connections that allow for the transformation of the muscles in the maxillofacial region under the influence of changes in autonomic tone. One of the proven factors activating sympathetic influences is acute or prolonged stress [42,43]. Today, it is associated with the occurrence of multiple cases of parafunctional activity of the masticatory muscles and the development of pathologies such as bruxism [44,45,46]. A study by Zhao Y.J. et al. noted increased glutamatergic transmission in the mesencephalic nucleus of the trigeminal nerve due to increased neuronal expression of VGLUT1 in a mouse model of chronic stress. The authors also noted an increase in acetylcholinesterase and creatine phosphokinase-MM in masticatory muscle tissue, indicating their hyperactivity [47]. A related study by the same author describes the influence of limbic system structures on masticatory activity under stress. Using neural mapping, the authors identified a significant role for projections between the amygdala and MesV in the development of masticatory muscle hyperactivity, confirmed by electromyographic studies [48,49].

Special attention should be paid to the effects of adrenaline and norepinephrine, which are the main mediators in the sympathetic nervous system. Schwarz P.B. et al. investigated the effect of noradrenergic transmission on trigeminal nerve motor neurons and the basal tone of the masticatory muscles. Using reverse microdialysis and neuropharmacological studies, it was possible to establish that exclusive activation of alpha-adrenergic receptors by norepinephrine does not lead to a change in muscle tone. However, in the presence of the active glutamatergic excitation of motor nuclei, norepinephrine significantly enhances this effect [50]. At the same time, there are also feedback relationships between the work of the trigeminal group of nuclei and norepinephrine. In their study, Ichikawa–Kato T. et al. assessed the effect of the disruption of occlusal relationships on the morphofunctional characteristics of the trigeminal nuclei and the level of norepinephrine in the brain after tooth extraction in rats. As a result, the authors demonstrated a decrease in the number of neurons in MesV and a decrease in norepinephrine concentrations in the hippocampus and cerebral cortex of rats, indicating a relationship between these two parameters with occlusal load and the functional activity of the masticatory muscles [51].

It is also worth considering the significance of the locus coeruleus, a nucleus that is part of the reticular formation and is the main source of norepinephrine biosynthesis in the central nervous system [52]. To date, sufficient data have accumulated describing the bilateral relationship between the locus coeruleus and the trigeminal nucleus system. Using immunohistochemistry, retrograde double labeling, and neural mapping, it was possible to confirm the role of this structure in organizing the antinociceptive system and chewing movements [53,54]. Liu Y. investigated the relationship between disturbances in masticatory muscle tone and the function of the locus coeruleus. While studying these parameters in rodents in a model of chronic stress caused by restricted mobility, the author noted a statistically significant difference in electromyographic parameters between the control and experimental groups. Mice exposed to the stress factor showed an increase in the amplitude of chewing waves and an increase in their frequency in the recording. At the same time, increased expression of markers of neuronal hyperactivity was detected in the locus coeruleus [55]. A number of researchers also report the significance of connections between the trigeminal nuclei and the locus coeruleus in neurodegenerative diseases. Changes in muscle tone and functional asymmetry of the trigeminal nerve may be risk factors for the development of neurodegeneration due to decreased activity of the locus coeruleus, where the first signs of the disease appear earliest [56,57,58].

## 5. The Influence of the Parasympathetic Division

**Figure 1:**
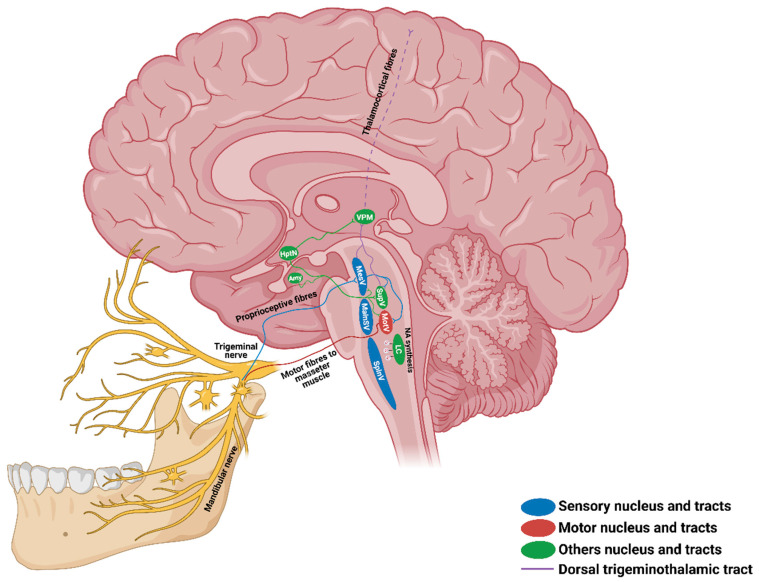
Structure of the proprioceptive reflex arc of the masseter muscles and autonomic influences on its structures. Note: MesV—sensory mesencephalic nucleus of the trigeminal nerve; MainSV—main sensory nucleus of the trigeminal nerve; SpinV—sensory spinal nucleus of the trigeminal nerve; MotV—motor nucleus of the trigeminal nerve; SupV—supratrigeminal nucleus; LC—locus coeruleus of the reticular formation with noradrenaline vesicles; Amy—amygdala; HptN—hypothalamic nuclei; VPM—ventral posterior medial nucleus of the thalamus. Thalamocortical fibers terminate in the cortex of the postcentral gyrus (description in the text). Created with BioRender.

When examining the parasympathetic division of the autonomic nervous system, it is worth highlighting some of its influence on masticatory muscle tone. The influence of parasympathetic innervation on hemodynamics in the masticatory muscles has been extensively studied in the scientific literature. Ishi H. et al. conducted a study examining the effect of electrical stimulation of the trigeminal nerve trunks on blood flow parameters in the masticatory muscles. An increase in blood flow was observed through stimulation of the trigeminal reflex, and the presence of parasympathetic fibers providing vasodilation was further confirmed [34]. In another study by this author, the effect of the electrical stimulation of parasympathetic fibers of other nerves on the vascular state of the masticatory muscles is examined. Following electrical stimulation, pronounced vasodilation was observed, confirmed by laser Doppler flowmetry data [59]. Additionally, the authors analyzed the interaction between the sympathetic and parasympathetic systems in the context of vascular tone. It was noted that simultaneous stimulation of sympathetic and parasympathetic fibers leads to vasoconstriction in the masticatory muscles. Based on this, it can be concluded that sympathetic influences are more potent, as their activation suppresses parasympathetic vasodilation. This is likely of great significance for stress states, in which a shift in balance toward sympathetic influences occurs. Changes in hemodynamic parameters may be a mechanism regulating metabolic processes in muscle fibers, which indirectly influences masticatory muscle tone [60]. Regarding the direct connection between parasympathetic influences and the trigeminal nuclei, few studies devoted to this topic have been identified. Robert C. et al. investigated the direct anatomical and functional connections between the centers of the parasympathetic nervous system and the nuclei of the trigeminal complex. They were able to discover that neurons of the paraventricular nuclei of the hypothalamus have direct connections with the sensory spinal nucleus of the trigeminal nerve [61]. This is not directly related to the regulation of masticatory tone, but can be considered as a potential link for the spread of parasympathetic impulses to other nuclei of the trigeminal complex (Figure 1). Thus, data on the precise mechanisms by which the parasympathetic division influences the state of the masticatory muscles remain somewhat limited. There is clear evidence regarding the effects of parasympathetic influences on the blood supply to the masticatory muscles and their connections with certain nuclei of the trigeminal complex. However, the question of whether the parasympathetic division has a direct effect on muscle tone requires further investigation.

## 6. The Relationship Between Psychoemotional State and Masticatory Muscle Function: Pathophysiological Aspects

According to the current literature, psychoemotional stress can alter masticatory muscle function by modulating neural connections, leading to increased activity of the masseter and temporalis muscles. Chronic stress may be a factor associated with the development of parafunctional activity, such as clenching and grinding [62,63].

Studies also show that stressful situations cause a significant increase in electromyographic (EMG) activity in the masticatory muscles [63,64,65]. The impact of stress on the masticatory muscles may be associated not only with increased functional muscle activity but also with its qualitative restructuring. For example, Angkulmahasuk et al., using surface electromyography (EMG) of the masseter muscle during wakefulness, analyzed the relationship between subjectively perceived stress on a visual analog scale (VAS) and muscle activity parameters: men with elevated stress levels recorded less frequent EMG bursts than women. The authors also noted that peak muscle activity decreased more significantly in men with elevated stress levels. People with low anxiety levels showed an increase in integrated muscle activity with increasing stress levels, while no similar effect was observed in people with moderate to high anxiety levels [66].

Referring to the data of Villada et al., it is necessary to note the absence of pronounced EMG modulation in response to stress in individuals with more severe anxiety. This may be a manifestation of less flexible motor responses to psychoemotional stress and, accordingly, a reduced ability to dynamically regulate the activity of the masticatory muscles [66,67].

Also worth mentioning is the study by Schmitter M., in which the authors noted that chronic stress increases the contraction frequency of individual fibers of the temporalis muscle [68]. The obtained data indicate that stress can modify the EMG activity pattern of the masticatory muscles, and the severity and direction of these changes are associated with individual psychological characteristics. Thus, chronic stress should be considered a factor capable, first and foremost, of transforming the bioelectrical (EMG) activity of the masticatory muscles and restructuring their activation pattern. Not only the short-term enhancement of muscle response but also the formation of stable motor habits becomes clinically significant.

In the context of nocturnal dental fractures, clinically significant conditions primarily involve those that result in repetitive occlusal loading during sleep and/or increase total muscle activity throughout the day. Therefore, the key form of cyclic masticatory muscle activity—sleep bruxism—and associated changes in masticatory muscles are discussed below [69].

Sleep bruxism is masticatory muscle activity during sleep, characterized as phasic (attacks recurring at regular intervals) or tonic (attacks manifest as prolonged, sustained muscle activity and do not recur at regular intervals), and is not a movement disorder or sleep disorder. Bruxism is characterized by clenching or grinding of the teeth and/or tense holding/forward protrusion of the mandible [69]. Although the authors distinguish only two types of sleep bruxism, instrumental studies (EMG) show that a combination of phasic and tonic components occurs in one episode [70]; this should be taken into account in the context of nocturnal tooth fractures because the combined variant increases the time and frequency of occlusal overload during sleep.

## 7. The Relationship Between Bruxism and Chronic Stress

According to a systematic review and meta-analysis by Chemelo, stress is associated with a higher likelihood of bruxism, but the level of evidence is assessed as insufficient [71]. In a recent review study by Pavlou, the author states that stress is frequently reported as an important contributing factor in the multifactorial etiology of bruxism [62]. In another cross-sectional study by González, the authors found a relationship between bruxism and stress in women, while no such association was observed in men [72]. Because this study was conducted in the format of an online survey based on subjective data, it is worth noting that the information obtained appears ambiguous, but may indicate potential patterns and form hypotheses for further research. In a study on the relationship between long-term stress and awake bruxism, Emodi–Perlman et al. concluded that bruxism and clenching can be considered mechanisms of adaptation to a long-term stress factor [73]. Thus, multiple lines of evidence indicate the existence of significant interrelations between chronic stress and the development of bruxism. At the same time, it can be stated unequivocally that stress is not the sole factor in the development of this condition. Genetic and neurological predispositions, medications, and a number of dental causes also play a major role.

## 8. The Relationship Between Bruxism and Tooth Fractures

Sleep bruxism is considered a form of increased activity of the masticatory muscles during sleep. From a clinical point of view, repetitive loads can be a factor in the destruction and damage of tooth structure [74,75]. However, it is important to understand that, with sleep bruxism, damage to the dental hard tissues is associated not with a single type of mechanical impact, but with a combination of several factors: repetitive cyclic occlusal load and the direction of force application [76,77]. Under conditions of non-axial occlusal load and/or parafunction, stress in the cervical region of the tooth, as a rule, increases. This should be taken into account in the context of fractures because the enamel in the area of the enamel–cementoenamel junction is represented by the thinnest layer, which creates conditions for the formation of a defect in the dental hard tissues precisely in this area [78,79]. Thus, the combination of parafunctional activity of the masticatory muscles and the anatomical vulnerability of the cervical zone can contribute to the development of microdamage to hard tissues with the subsequent formation of clinically significant defects. These primarily include pathological abrasion, enamel cracks, abfraction (wedge-shaped defects), and tooth fractures [80]. Importantly, modern studies evaluate such changes within the context of the clinical picture of bruxism and signs of occlusal overload. This allows us to consider the identified defects in the dental hard tissues as clinically significant manifestations of parafunctional stress.

## 9. Clinical Manifestations: Tooth Wear, Cracks, and Fractures

**Figure 2:**
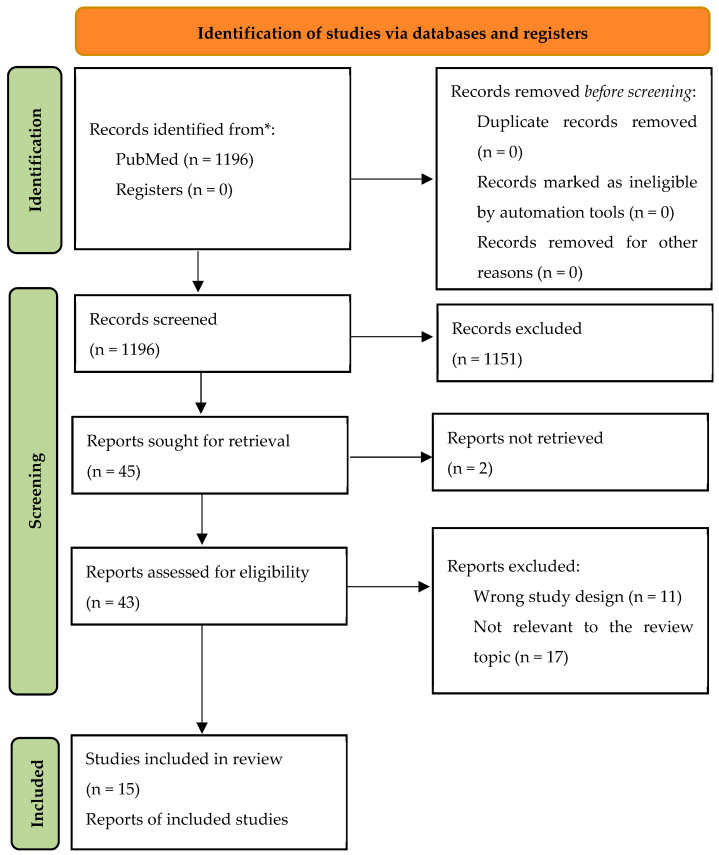
Identification of studies via databases and registers. * Keywords combination (bruxism OR “sleep bruxism”) AND (“masticatory muscles” OR masseter OR temporalis).

To provide a clear illustration of the data on how abnormalities in masticatory muscle tone impact the condition of hard dental tissues, they were organized in the form of a table (Table 1). A comprehensive review was conducted in the MEDLINE, PubMed, Web of Science, and Scopus databases for papers published in the English language. Out of 1196 eligible papers, 15 were included for final appraisal (Figure 2).

One of the first clinical manifestations of the consequences of bruxism is tooth wear. In a study by Laksamikeeratikul I. et al., which included 16 patients with an average age of about 28 years, the authors noted a statistically significant difference in the wear of the occlusal surface and incisal edges of all teeth in the group with bruxism compared to the control group [82]. A study by Wetselaar P. confirms these data and indicates the presence of enamel wear in patients with sleep bruxism [83]. At the same time, in a review analysis by Bronkhorst H., the authors examined 30 publications devoted to the relationship between bruxism and tooth wear and came to the conclusion that the results are ambiguous: some studies did not find or found a weak relationship between tooth wear and bruxism [81]. Data from more recent clinical observations suggest that a relationship between bruxism and tooth wear exists, but requires interpretation taking into account associated factors, primarily age: in a cross-sectional clinical study by Popescu A., the authors analyzed the effects of excessive occlusal load and noted that tooth wear is associated with bruxism and can be considered a clinical sign of parafunctional activity of the masticatory muscles [80].

As mechanical stress progresses, the clinical picture becomes more pronounced: enamel and dentin cracks may appear. Cracks are incomplete tooth fractures that typically damage enamel and dentin, and sometimes also the pulp and periodontal tissue [84]. In a clinical review by Mylonas P., it is noted that, in bruxism, occlusal loads can reach 900 kg, and such indicators are considered potentially significant in the formation of tooth cracks [85]. In another case–control study, Zhang A. and colleagues noted that changes in bite may disrupt occlusal contacts, leading to increased loads and a higher risk of cracks [86]. Although this study does not consider bruxism as an etiological factor, its results confirm the importance of occlusal overload in the pathogenesis of tooth cracks.

In a review article by Li F., the authors mention that bite force can influence the occurrence of cracks and the destruction of tooth structure [87]. Modern publications more frequently describe fractures and other more severe damage to hard dental tissues, which are assessed as possible consequences of parafunctional activity of the masticatory muscles. In a clinical cross-sectional study, Popescu and colleagues note that tooth fractures were more common in patients with bruxism than in those who do not suffer from this condition [80]. In another long-term study, the authors examined the relationship between parafunctional activity of the masticatory muscles, maximum bite force, and tooth/ceramic veneer fractures. The following results were obtained: in patients with bruxism, the maximum clenching force was statistically significantly higher, and the proportion of tooth and ceramic fractures per total number of teeth/orthopedic units was higher than in patients without clinically confirmed bruxism [75]. In a clinical case format, Tunçekin and authors describe a 35-year-old patient who developed sleep bruxism after starting duloxetine treatment, and a few weeks later, a fracture of the right upper first molar, which had previously been endodontically treated, was recorded, as well as a partial fracture of a tooth in the same area in the mandible [88]. In addition to all the above-mentioned data, it should be noted that the course of the pathological process also depends on associated factors. One such factor is patient age. It is known that, over the course of a person’s life, loss of hard dental tissues occurs under the influence of masticatory load. This process is termed physiological wear and represents a normal response of the hard dental tissues to adequate mechanical load. However, in elderly individuals, when bruxism is superimposed on physiological wear, a cumulative effect occurs. This manifests as an increased loss of hard dental tissue and exacerbates the course of the pathological process [89]. Particular attention should also be paid to occlusal relationships. Although, according to various sources, occlusal anomalies cannot be the primary cause of bruxism, some evidence indicates that they may accelerate the rate of hard dental tissue wear [90,91]. Premature contacts are of particular importance in this regard, as their presence may aggravate the parafunctional activity of the masticatory muscles [92]. This confirms the need for the orthodontic correction of occlusal anomalies to prevent serious complications [93]. Furthermore, a number of morphological features of the maxillofacial region may also contribute to the progression of bruxism complications [94].

## 10. Preventive Measures

To prevent the development of dental hard tissue pathology, a number of treatment and prophylactic methods are currently used, aimed at reducing or redistributing abnormal masticatory pressure. Some of these methods involve drug therapy, which suppresses various central neurogenic influences on masticatory muscle tone. These include drugs from the groups of antidepressants, benzodiazepines, adrenergic blockers, antiepileptic drugs, and others [95,96,97,98]. However, the evidence base for the effectiveness of drug therapy is still insufficient, which dictates the conditions for conducting new research in this area [99]. The most commonly used methods are botulinum toxin type A injections and the use of dental mouth guards [100,101]. The former limit synaptic transmission at the neuromuscular synapse by disrupting the release of neurotransmitters, which leads to relaxation of the masticatory muscles. Numerous studies have demonstrated the ability of intramuscular injections of botulinum toxin to reduce maximum occlusal pressure, the frequency of bruxism attacks, and their consequences [102,103,104,105]. Occlusal splints made of polymethyl methacrylate and acrylic can reduce occlusal load and decrease tension in the masticatory muscles [106,107]. At the same time, it should be noted that there is a considerable body of research indicating the controversial efficacy of mouthguard use in bruxism. Evidence suggests that standard mouthguards do not reduce muscle tone, as confirmed by electromyography data [108]. A systematic review by Riley P. et al. confirms the ambiguity of data regarding the clinical efficacy of splint therapy. This underscores the need for further investigation of this issue through randomized clinical trials [109]. Various preventive measures aimed at correcting the stress factor have also been noted. Among such interventions, cognitive behavioral therapy stands out as a primary approach; according to some data, it reduces the frequency of bruxism episodes and enhances the effectiveness of occlusal mouthguards [110,111]. Additional data indicate that sleep quality plays an important role in bruxism. A. Tuncer et al., in their study, demonstrate that an 8-week course of sleep hygiene therapy in children is an effective method for the prevention and treatment of bruxism [112]. The literature also contains data on the use of neurobiomodulation techniques and emotional behavior modification via mobile applications. These methods can reduce brain electrical activity and correct plasma cortisol levels, but evidence regarding their effects on the frequency of bruxism episodes and their manifestations remains limited [113,114]. Additionally, C. Restrepo–Serna et al. report a reduction in the frequency of sleep bruxism in children when limiting screen time and the intake of fast-digesting carbohydrates [115].

## 11. Discussion and Conclusions

Accumulated knowledge about the relationships between the functioning of various parts of the central nervous system under chronic stress and masticatory muscle activity allows us to conceptualize changes in muscle tone as a complex process controlled by multiple structures and endogenous factors. A key role in this process is played by changes in the activity of trigeminal nucleus neurons under the influence of subcortical structures and major autonomic centers. These can exert both direct and indirect (via metabolic and circulatory changes) effects on muscle tone. Therefore, in further studies of this condition, the state of autonomic tone should be examined in detail, as alterations in autonomic tone may have a significant impact on masticatory muscle tone. This is particularly relevant for sympathetic influences, which can intensively affect the trigeminal complex nuclei, neurotransmitter metabolism, and vascular tone. Additionally, it has been determined that sympathetic influences dominate over comparable parasympathetic influences with respect to muscle tone. Current data allow us to consider bruxism, one of the manifestations of the disruption of muscle tone physiology, as a clinically significant factor in occlusal overload, contributing to the development of hard dental tissue damage both at night and during the day. The most common types of damage in this context are pathological wear and the formation of cracks. Tooth fractures are less frequent but also occur within the pathological presentation of the disease. This is supported by studies evaluating bruxism in relation to signs of excessive occlusal force and the increased frequency of damage associated with excessive parafunctional activity of the masticatory muscles. However, the currently available evidence base remains somewhat limited. Many publications consider bruxism as a potential etiological factor rather than the sole established cause, due to the multifactorial nature of tooth fractures (tooth morphology, occlusal contacts, the condition of restorations, associated loads, etc.). Furthermore, there are relatively few clinical cases in the modern literature where tooth fractures are described solely in the context of bruxism. Associated conditions are of great importance. It is often the combined influence of several previously discussed factors together with bruxism that shapes the characteristic clinical and pathological picture. In contrast, the effect of parafunctional activity of the masticatory muscles alone may have milder manifestations. While there are currently a number of methods for preventing dental pathology associated with bruxism, these methods are not always highly effective. This underscores the need for further clinical research aimed at clarifying how the parafunctional activity of the masticatory muscles impacts the pathology of dental hard tissues and the maxillofacial region as a whole.

## 12. Future Research Necessities

Despite considerable attention to the problem of bruxism, many aspects of this condition remain incompletely understood. To improve understanding of this condition, future research should focus on the neurobiological underpinnings of bruxism. Investigating interactions at the level of neuromuscular synapses and studying features of the connectome could make a substantial contribution to this topic. With regard to dental problems, additional attention should be paid to the relationships between bruxism and other factors involved in hard dental tissue pathology. Which of these factors may potentiate the effect of increased occlusal load, and which may not? Furthermore, the development of new preventive measures represents a key direction for future research. As studies indicate, many existing and actively evolving methods show good results in managing the consequences of bruxism.

## Figures and Tables

**Table 1:** Characteristics of included studies.

	Authors	Research Design	Sample Size	Country of Study	Description of Results
Bruxism and direct and indirect restorations failure: A scoping review [74]	Tanya Al-Talib, Xan Goodman, Hassan Ziada, Neamat Hassan Abubakr	Scoping review	46 included studies	USA	Most included studies demonstrated that bruxism was associated with an increased risk of the failure of direct and indirect restorations, whereas the most favorable survival outcomes were observed for monolithic zirconia and lithium disilicate (LiDi) restorations.
Relationship between bite force, bruxism, and fractures of teeth and dental restorations [75]	Bruno Ramos Chrcanovic, Tom Bergengren, Nikola Stanisic, Sahar Sohrabi, Christel Larsson, Peter Svensson, Birgitta Häggman-Henrikson	Retrospective cohort follow-up study	51 implant-treated patients: 30 probable bruxers and 21 matched controls	Sweden	The results demonstrated that individuals with bruxism exhibited a statistically significantly greater mean jaw-clenching force compared with the control group.
Fatigue and wear of human tooth enamel: A review [76]	Jamie J Kruzic, Mark Hoffman, Joseph A Arsecularante	Review	N/A	Australia	Cyclic loading associated with bruxism contributes to the development of fatigue cracks, attritional wear, and enamel degradation, while the processes of enamel fatigue and wear are closely interconnected.
Influence of occlusal loading type and cyclic fatigue on the mechanical behavior of sound maxillary premolars: a laboratory and 3D finite element analysis [77]	Alexandre Coelho Machado, Paulo Vinícius Soares, Christian de, Almeida Soares, Bruno Rodrigues Reis, Lívia Fávaro Zeola, Luís Henrique Araújo Raposo	In vitro laboratory study with 3D finite element analysis	30 sound human maxillary premolars	Brazil	Non-axial cyclic occlusal loading significantly increased stress concentration, deformation, and fatigue damage to cervical enamel and dentin, confirming the role of occlusal stress in the development of non-carious cervical lesions.
Enamel thickness after preparation of tooth for porcelain laminate [78]	Ayoub Pahlevan, Mansoreh Mirzaee, Esmaeil Yassine, Ladan Ranjbar Omrany, Masumeh Hasani Tabatabaee, Sakineh Arami, Mehdy Abbasi		Part I: 20 extracted maxillary incisors Part II: 30 maxillary central incisors	Iran	The authors investigated enamel thickness in different regions of the labial surface of maxillary incisors and evaluated the effect of chamfer and knife-edge preparations for porcelain laminate veneers on the risk of dentin exposure. The minimum enamel thickness was found in the cervical third of the tooth, while chamfer preparation was associated with a significantly higher risk of dentin exposure compared with knife-edge preparation.
Lesion morphology modification is unnecessary for non-carious cervical lesion restorations: a comparison of stiff and flowable composites [79]	Phetcharat Dhammayannarangsi, Sorapon Na Lampang, Pimpichaya Traithipsirikul, Aoraya Supamongkol, Pat Kittipongphat, Vorapat Trachoo, Thanaphum Osathanon, Sontipee Aimmanee, Vincent Everts, Lakshman Samaranayake, Nuttapol Limjeerajarus, Chalida Nakalekha Limjeerajarus		15 sound maxillary first premolars group, 15 sound maxillary first premolars, horizontal–oval–round of non-carious cervical lesion group, 15 sound maxillary first premolars, horizontal–oval–wedge of non-carious cervical lesion group		
Oral clinical and radiological signs of excessive occlusal forces in bruxism [80]	Adrian Marcel Popescu, Mihaela Ionescu, Sanda Mihaela Popescu, Alin Gabriel Ionescu, Diana Elena Vlăduțu, Monica Mihaela Iacov-Crăițoiu, Alexandru Ștefârță, Luana Corina Lascu, Veronica Mercuț	Cross-sectional clinical study	181 patients	Romania	According to the study results, patients with bruxism demonstrated a statistically significantly higher prevalence of masseter muscle hypertrophy, increased tooth wear, abfraction, dental fractures, and bone apposition at the mandibular angles, indicating the impact of excessive chronic occlusal loading on the hard dental tissues.
Tooth wear and bruxism: A scoping review [81]	Hilde Bronkhorst, Stanimira Kalaykova, Marie-Charlotte Huysmans, Bas Loomans, Tatiana Pereira-Cenci	Scoping review	30 included studies	Netherlands	Most studies demonstrated absent or weak associations between tooth wear and bruxism, with the exception of studies evaluating cervical tooth wear. Associations were reported more frequently when bruxism was assessed by self-report, whereas studies employing multivariate analyses generally failed to identify a significant relationship, except in investigations of cervical wear.
Digital measurement of tooth wear in sleep bruxism patients wearing occlusal splints [82]	Irene Laksamikeeratikul, Supawadee Jariyasakulroj, Thiprawee Chattrattrai, Sunee Pongrojpaw	Prospective cohort study	16 patients	Thailand	In patients with sleep bruxism, progression of the wear of the occlusal surfaces and incisal edges continued despite the use of nighttime occlusal splints, indicating persistent chronic mechanical loading on the hard dental tissues.
Associations between tooth wear and dental sleep disorders: A narrative overview [83]	Peter Wetselaar^,^ Daniele Manfredini, Jari Ahlberg, Anders Johansson, Ghizlane Aarab, Chryssa E Papagianni, Marisol Reyes Sevilla, Michail Koutris, Frank Lobbezoo	Narrative overview	101 included studies	International	The authors reported an association between sleep bruxism and mechanical tooth wear, while emphasizing the multifactorial nature of tooth wear development.
Cracked tooth syndrome: A report of three cases [84]	Kadandale Sadasiva, Sathishmuthukumar Ramalingam, Krishnaraj Rajaram, Alagappan Meiyappan	Case series	3 patients	India	Cracked tooth syndrome was more common in patients aged 30–50 years and was associated with large restorations, loss of hard tooth tissue, and other predisposing factors; the prognosis depended on the depth and extent of the crack.
Bruxism and endodontics: how to manage cracked teeth [85]	Petros Mylonas, Ivy Gitonga, Kostas Ioannidis	Narrative review with case reports	Several clinical cases presented	UK	The authors noted that the prognosis of cracked teeth depends on the depth and extent of the crack, and controlling bruxism and occlusal overloading is important for tooth preservation.
Symptomatic cracked teeth: Associations with patient-level and tooth-level factors-A case–control study [86]	Aidi Zhang, Zhuping Sang, Xige Zhang, Yu Yang, Zhe Yang, Wei Wang	Case–control study	100 patients	China	The authors found that cracked teeth were more common in patients aged 30–39 years and were associated with hard food consumption, unilateral chewing, deep overbite, dental caries, and occlusal tooth wear. Additionally, certain molar cusp inclinations were associated with an increased risk of cracked teeth.
Review of Cracked Tooth Syndrome: Etiology, Diagnosis, Management, and Prevention [87]	Fei Li, Yaoyao Diao, Jiayin Wang, Xingyu Hou, Shuzhan Qiao, Jiawen Kong, Yunhan Sun, Eui-Seok Lee, Heng Bo Jiang	Narrative review	N/A	China, Republic of Korea	The authors emphasized the role of bruxism and occlusal overloading in the development of tooth cracks, as well as the importance of early diagnosis and timely treatment to tooth preservation.
Duloxetine-Induced Sleep Bruxism and Tooth Fracture in Fibromyalgia [88]	İsmail Tunçekin, Murat Toprak	Case report	1 patient	Turkey	In the presented clinical case, the development of sleep bruxism during duloxetine therapy was accompanied by tooth fracture, which may indicate the negative impact of chronic parafunctional loading on the hard dental tissues.

## Data Availability

No new data were created or analyzed in this study.
